# Game of thrones among AUXIN RESPONSE FACTORs—over 30 years of MONOPTEROS research

**DOI:** 10.1093/jxb/erad272

**Published:** 2023-08-05

**Authors:** Barbara Wójcikowska, Samia Belaidi, Hélène S Robert

**Affiliations:** Mendel Centre for Genomics and Proteomics of Plants Systems, CEITEC MU - Central European Institute of Technology, Masaryk University, Brno, Czech Republic; Institute of Biology, Biotechnology, and Environmental Protection, Faculty of Natural Sciences, University of Silesia in Katowice, Katowice, Poland; Mendel Centre for Genomics and Proteomics of Plants Systems, CEITEC MU - Central European Institute of Technology, Masaryk University, Brno, Czech Republic; National Centre for Biomolecular Research, Faculty of Science, Masaryk University, Brno, Czech Republic; Mendel Centre for Genomics and Proteomics of Plants Systems, CEITEC MU - Central European Institute of Technology, Masaryk University, Brno, Czech Republic; Institute of Science and Technology Austria (ISTA), Austria

**Keywords:** *Arabidopsis thaliana*, auxin, embryogenesis, flowering, meristem, plant, transcription factor, vascularization

## Abstract

For many years, research has been carried out with the aim of understanding the mechanism of auxin action, its biosynthesis, catabolism, perception, and transport. One central interest is the auxin-dependent gene expression regulation mechanism involving AUXIN RESPONSE FACTOR (ARF) transcription factors and their repressors, the AUXIN/INDOLE-3-ACETIC ACID (Aux/IAA) proteins. Numerous studies have been focused on MONOPTEROS (MP)/ARF5, an activator of auxin-dependent gene expression with a crucial impact on plant development. This review summarizes over 30 years of research on MP/ARF5. We indicate the available analytical tools to study *MP/ARF5* and point out the known mechanism of MP/ARF5-dependent regulation of gene expression during various developmental processes, namely embryogenesis, leaf formation, vascularization, and shoot and root meristem formation. However, many questions remain about the auxin dose-dependent regulation of gene transcription by MP/ARF5 and its isoforms in plant cells, the composition of the MP/ARF5 protein complex, and, finally, all the genes under its direct control. In addition, information on post-translational modifications of MP/ARF5 protein is marginal, and knowledge about their consequences on MP/ARF5 function is limited. Moreover, the epigenetic factors and other regulators that act upstream of *MP/ARF5* are poorly understood. Their identification will be a challenge in the coming years.

## Teaser

A review of the action of MP/ARF5 in auxin-dependent regulation of gene expression, its role in the plant developmental process, and available tools for molecular analysis.

## Introduction

The name auxin comes from the Greek word ‘auxein’, meaning ‘growth’. The first reports of auxin date back to 1880, when Charles and Francis Darwin hypothesized the existence of ‘a growth-regulating stimulus’ controlling the process of phototropism (Darwin and Darwin, 1897). Darwin proposed that the ‘stimulus’, though he could not identify it, moves from the site of stimulus perception to the area of responsibility, the bending site. In 1926, Fritz Went isolated a chemical compound affecting the elongational growth of *Avena sativa* seedlings lacking a shoot apical meristem (SAM; Went, 1926). A few years later, the Fritz Kögl team isolated and identified indole-3-acetic acid (IAA) from human urine (Kögl *et al.*, 1934). Larry Vanderhoef and his group in 1976 suggested that the initial response to auxin is driven by a rapid process, whereas a second response involves gene expression (Vanderhoef *et al*., 1976). The first reports of auxin’s potential influence on the transcriptional regulation of many genes appeared in the 1980s (reviewed in Guilfoyle and Key, 1986; Theologis, 1986). Since then, the development of molecular techniques and the use of model plants have led to the identification of many genes involved in auxin metabolism, perception, and transport (Paciorek and Friml, 2006; Quint and Gray, 2006; Calderon-Villalobos *et al.*, 2010).

The process of auxin biosynthesis in plants is not fully understood, and intensive research is still underway to uncover and understand all the enzymes involved. Two pathways have been identified for the synthesis of endogenous auxin IAA, one dependent on and one independent of l-tryptophan (l-Trp). The l-Trp-independent pathway is poorly understood, in contrast to the l-Trp-dependent pathway, for which many enzymes have already been identified. The l-Trp-mediated IAA biosynthesis pathway can be further divided into four routes. In the first route, tryptophan aminotransferases [TRYPTOPHAN AMINOTRANSFERASE OF ARABIDOPSIS 1 (TAA1) and TAA1-RELATED 1-2 (TAR1-TAR2)] synthesize indolyl-3-pyruvic acid (IPyA) from l-Trp which is converted into IAA by the flavin monooxygenases YUCCA (YUC1–YUC11) (Stepanova *et al.*, 2008, 2011). This mode of IAA synthesis is common in the plant world (Zhao, 2012). Another auxin biosynthesis pathway requires tryptophan oxidase activity to produce indolyl-3-acetaldoxime (IAAox). IAAox can be converted into indolyl-3-acetonitrile (IAN), indolyl-3-acetamide (IAM), and indolyl-3-methyl glucosinolates. The hydrolysis of IAN, probably under the influence of nitrile aminohydrolases (NITRILASE 1–4, NIT1–NIT4), produces IAA (Lehmann *et al.*, 2017). IAN can transform into IAM catalyzed by NITs (Sugawara *et al.*, 2009). l-Trp is also directly converted to IAM. Lastly, IAM can be hydrolyzed by indolyl-3-acetamide hydrolase (AMI1) into IAA (Pollman *et al.*, 2006; Novák *et al.*, 2012).

Proteins facilitating the transport of IAA between and within cells are well known and well characterized. Among them, plasma membrane-localized AUX1, LIKE-AUX1 (LAX), and NITRATE TRANSPORTER 1.1 (NRT1.1) proteins import IAA into the cell. IAA moves inside the cell, in and out the endoplasmic reticulum, and exits the cell mainly via the activity of PIN-FORMED (PIN) and PILS (PIN-LIKE) auxin exporters. Members of the ARABIDOPSIS THALIANA ATP-BINDING CASSETTE B (ABCB) family can mediate auxin influx and efflux (reviewed by Barbosa *et al.*, 2018).

**Fig. 1.**
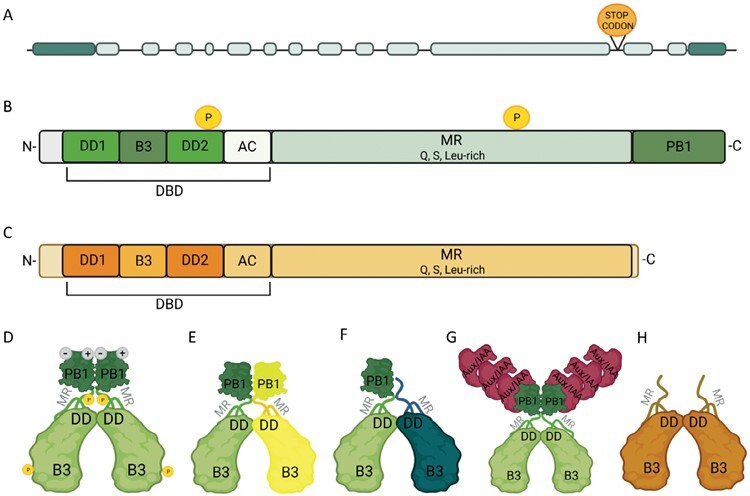
Structure of the *MP/ARF5* gene. Dark green boxes, the 5ʹ- and 3ʹ-untranslated regions; lines, introns; light green boxes, exons (A). Domains present in canonical MP/ARF5 protein (B) and its isoform MP11ir (C) involved in auxin-dependent and independent regulation of gene expression; DBD, DNA-binding domain; MR, middle region domain; PB1/III-IV, Phox/Bem1/domain III and IV; DD, dimerization domin; B3, B3 DNA-binding domain; AC, ancillary subdomain; Q, glutamine; S, serine; L, leucine; P, phosphoryl group. Possible interactions of MP/ARF5, MP11ir with ARF, and Aux/IAA proteins (D–H). MP/ARF5–MP/ARF5 homodimer (D); heterodimer MP/ARF5–canonical ARFs (E); heterodimer MP/ARF5–truncated ARFs (F); MP/ARF5–Aux/IAA complex (G); homodimer of truncated MP11ir isoform (H). Created with BioRender.com.

During more than three decades of intensive research on auxin, four groups of IAA receptors have been identified: membrane- and apoplast-localized AUXIN BINDING PROTEIN 1 (ABP1) together with its plasma membrane-localized partner TRANSMEMBRANE KINASE (TMK1–TMK4), TRANSPORT INHIBITOR RESPONSE 1 (TIR1), and its homologs AUXIN SIGNALING F-boxes (AFB1–AFB5) localized in the nucleus and cytosol, and nuclear-located S-PHASE KINASE-ASSOCIATED PROTEIN 2a (SKP2a), and ETTIN/AUXIN RESPONSE FACTOR 3 (ETT/ARF3) (reviewed by Yu *et al.*, 2022). The central role of the nuclear auxin perception mechanism is transcriptional reprogramming, conducted by AUXIN/INDOLE-3-ACETIC ACID (Aux/IAA) and AUXIN RESPONSE FACTOR (ARF) proteins. Aux/IAA repressors are ubiquitinated in the presence of auxin and subsequently degraded, relieving the repression on ARF-targeted loci. In the Arabidopsis genome, 23 *ARF* genes and 29 *Aux/IAA* repressors were identified (Ulmasov *et al*., 1997b; Guilfoyle and Hagen, 2001; Liscum and Reed, 2002). Among ARFs, ARF5, also called MONOPTEROS (MP) or INDOLE-3-ACETIC ACID INDUCIBLE 24 (IAA24), was one of the first identified (Hardtke and Berleth, 1998). MP/ARF5 is a transcription factor (TF) that regulates the expression of targeted genes in an auxin dose-dependent manner (Mattsson *et al.*, 2003; Krogan *et al.*, 2014). The *monopteros* (*mp*) mutant was discovered after screening a population of Landsberg erecta (Ler) *Arabidopsis thaliana* mutants obtained by ethyl methanesulfonate (EMS) chemical mutagenesis (Mayer *et al.*, 1991). The *MP/ARF5* locus and sequence in chromosome 1 were characterized in detail by Hardtke and Berleth (1998). The Arabidopsis *MP/ARF5* gene is 4736 bp long, consisting of 13 exons (Fig. 1A). MP/ARF5 belongs to class A-ARF (together with ARF6-9 and ARF19), the best-studied ARF group (Guilfoyle and Hagen, 2007; Finet *et al.*, 2013). Although MP/ARF5 in model plants such as Arabidopsis has been well characterized, its identities and potential roles in non-model plants are less studied. With increasing numbers of sequenced plant genomes, the *ARF* family and the *MP/ARF5* gene have been identified at the whole-genome level in Arabidopsis (Okushima *et al.*, 2005) and in many plant species. The knowledge about *MP/ARF5* action is being slowly explored in commercially important species (Xu *et al.*, 2019; Yue *et al.*, 2020; Mei *et al.*, 2022). Transferring knowledge about MP/ARF5 function to non-model plants of important agro-botanical and industrial significance is a challenge for the coming years, requires further studies, and may help better understand MP/ARF5 action.

## Evolution and origin of the nuclear auxin response pathway

A nuclear auxin response is conserved across land plants and requires the presence of the three major auxin signaling proteins, ARFs, Aux/IAAs, and TIR1/AFBs. These three signaling components appeared in and are limited to land plants (Lavy and Estelle, 2016; Leyser, 2018; Mutte *et al*., 2018). Phylogenomic analyses and genetic approaches revealed that bryophytes, including many model species such as *Physcomitrium patens* for the mosses, *Selaginella moellendorffii* for the lycophytes (Floyd and Bowman, 2007; Rensing *et al*., 2008), and *Marchantia polymorpha* for the liverworts (Flores-Sandoval *et al*., 2015; Kato *et al*., 2015), possess all the canonical elements, with all the conserved domains necessary for the nuclear auxin pathway (Kato *et al*., 2017), and act similarly to the auxin signaling machinery in angiosperms (Flores-Sandoval *et al*., 2015, 2016; Kato *et al*., 2015, 2017, 2018). Fewer gene copies for the canonical components (ARFs, Aux/IAAs, TIR1/AFBs, and TPL) are found in bryophytes compared with angiosperms (Paponov *et al*., 2009; Flores-Sandoval *et al*., 2015; Kato *et al*., 2015; Lavy *et al*., 2016).

Some algae are sensitive to the application of exogenous IAA (Ohtaka *et al*., 2017), and auxin-like core elements exist in different algal species (Cooke *et al*., 2002; Stirk *et al*., 2002). However, no clear evidence demonstrated that algae have an ARF–Aux/IAA complex mediating the auxin signaling pathway as in land plants (Lau *et al*., 2009). For example, neither Aux/IAA nor TIR/AFB proteins were detected in red algae. Still, an ARF-like structure was found, with 20–30 conserved amino acids replacing the B3 DNA-binding domain and a C-terminal bromodomain instead of the Phox/Bem1 (PB1) domain (Mutte *et al*., 2018). Charophyte ARFs have all the characteristic functional subdomains, but the Aux/IAAs are non-canonical, lacking the degron domain (Mutte *et al.*, 2018). In land plants, ARFs are classified into A, B, and C classes (Finet *et al*., 2013). Many charophytes encode two groups of ARFs, group C and a combined A/B group (Mutte *et al*., 2018; Martin-Arevalillo *et al*., 2019). The three ARFs classes were probably derived from a common ancestral proto-ARF protein with class C-like characteristics (Martin-Arevalillo *et al.*, 2019). Taken together, the ancestral genes encoding all the auxin core components required for a functional nuclear auxin response, including auxin biosynthesis, transport, and signaling, were present, established, and conserved in all terrestrial plants (Flores-Sandoval *et al*., 2015; Bowman *et al*., 2017, 2019; Mutte *et al*., 2018) and evolved from an algal auxin-independent pathway ancestor (Martin-Arevalillo *et al*., 2019).

## Structure of MP/ARF5 protein

The canonical MP/ARF5 protein contains 902 amino acids with a mol. wt of 99.65 kDa. In addition to the canonical protein, two isoforms were identified in Arabidopsis: ARF5ʹ with unknown function (891 amino acids, 98.45 kDa) (TAIR; https://www.arabidopsis.org/) and MP11ir (815 amino acids, 89.94 kDa) (Cucinotta *et al.*, 2021). The canonical MP/ARF5 protein is composed of three main domains: the N-terminal DNA-binding domain (DBD), the middle region (MR) domain, and the Phox/Bem1 (PB1) known as a C-terminal domain (previously called domain III and IV) (Cancé *et al.*, 2022) (Fig. 1B). The MP11ir isoform lacks a C-terminal PB1 domain (Fig. 1C).

The DBD consists of three subdomains: the B3 DNA-binding motif flanked by two dimerization domains (DDs) and, at the C-terminus of the DBD domain, an ancillary subdomain (AC), related to the Tudor domain (a five-stranded β-barrel-like structure). The AC subdomain interacts tightly with the DD subdomains. However, its exact function remains unknown (Boer *et al.*, 2014). Furthermore, the DD mediates homodimerization (Fig. 1D) of MP/ARF5 and heterodimerization with other ARF proteins (Fig. 1E, F). MP/ARF5 can act as a monomer and dimer. Homodimerization is necessary for its action and binding to the promoter of its target genes, and may define MP/ARF5–DNA binding specificity *in vivo* (Boer *et al.*, 2014; Fontana *et al.*, 2023). However, the role of potential heterodimers is still under discussion (Cancé *et al.*, 2022; Caumon and Vernoux, 2023). MP/ARF5 binds to DNA via a single B3 DNA-binding domain, a plant-specific domain of ~110 amino acids. The B3-type TF superfamily contains 118 members in Arabidopsis, having functions mainly related to hormone response, and includes the ARFs, LEC2-ABI3-VAL (LEAFY COTYLEDON 2-ABSCISIC ACID INSENSITIVE 3-VP1-ABI3-LIKE, LAVs), RELATED TO ABI3/VP1 (RAVs), and REPRODUCTIVE MERISTEM (REM) (Yamasaki *et al.*, 2013). The B3 domain is essential for MP/ARF5 binding to auxin-responsive elements (AuxREs) in auxin-induced gene promoters. It has been demonstrated by DNA affinity purification sequencing (DAP-seq) that the hexanucleotide-target motif 5ʹ-TGTCGG-3ʹ rather than the canonical AuxRE 5ʹ-TGTCTC-3ʹ is the preferred binding sequence for MP/ARF5 (O’Malley *et al.*, 2016; Freire-Rios *et al.*, 2020). A ChIP-seq analysis showed an overlap of target genes between three positive ARFs [MP/ARF5, ARF6, and NON-PHOTOTROPHIC HYPOCOTYL 4 (NPH4)/ARF7] and one repressor ARF10 (Xie *et al.*, 2022, Preprint). It suggests that ARFs may compete for binding to AuxREs, depending on the cell types. The DBD of MP/ARF5 can recognize three combinations of two AuxRE motifs when located in different DNA strands, facing each other (inverted repeats, IRs), the same strand, in the same direction, one after the other (direct repeats, DRs), or different DNA strands back-to-back (overturned repeats, ERs). Additionally, MP/ARF5 binds with a strong affinity to two AuxREs separated with specific spacing, namely IR7–8 and IR18; DR4–5, DR14–16, and DR25; and ER3, ER13, and ER23, where the numbers indicate the number of bases between two AuxREs (Stigliani *et al.*, 2019; Freire-Rios *et al.*, 2020).

The sequence of the amino acid residues of the MR domain is specific for each ARF TF and determines whether the TF acts as a transcriptional activator or repressor (Li *et al.*, 2016; Powers and Strader, 2020). In the case of MP/ARF5, ARF6–ARF8, and ARF19 proteins, their MR domain is rich in amino acids such as glutamine, serine, and leucine, implying that they belong to the activators (Tiwari *et al.*, 2003; Guilfoyle and Hagen, 2007). This was confirmed in Arabidopsis (Tiwari *et al.*, 2003), rice (Shen *et al.*, 2010), and tomato (Zouine *et al.*, 2014). The MR domain of the repressor ARFs (ARF1–ARF4, ARF9–ARF18, and ARF20–ARF22) is enriched in serine, proline, leucine, and glycine (Ulmasov *et al*., 1997a; Guilfoyle and Hagen, 2007). MP/ARF5 can be phosphorylated on Thr163 in the DBD and Ser647 in the MR (Fig. 1B, D) by interacting with a glycogen synthase kinase BIN2-LIKE 1 (BIL1). This phosphorylation attenuates the interaction between MP/ARF5 and Aux/IAA repressors, thus enhancing MP/ARF5 activity (Han *et al.*, 2018).

Lastly, the PB1 domain, at the C-terminal end of the MP/ARF5 protein, provides positive and negative electrostatic interfaces for directional protein interaction (Korasick *et al.*, 2014). MP/ARF5 cooperates with short-lived nuclear repressors, Aux/IAA proteins, through this domain. Computer simulations showed that MP/ARF5 could interact with almost all Aux/IAA proteins (excluding IAA6, IAA9, and IAA26), and *in vitro* analyses confirmed the interaction between MP/ARF5 and IAA1, IAA3, IAA13–IAA14, IAA17, and IAA19 (Vernoux *et al.*, 2011) (Fig. 1G). The PB1 domain also contributes to the dimerization and condensation of A-type ARF factors (Jing *et al.*, 2022). Indeed, deletion of the PB1 domain lowers ARF–ARF interaction affinities (Nanao *et al.*, 2014; Powers *et al.*, 2019).

The MP/ARF5 isoform, MP11ir, lacking the PB1 domain, is produced by RNA alternative splicing during ovule development. The retention of intron 11 in the transcript results in a stop codon in the preserved intron (Fig. 1A), leading to the translation of a truncated protein (Fig. 1C, H). By polysome profiling using pre-fertilization inflorescences, it was suggested that *MP11ir* transcripts might escape nonsense-mediated mRNA decay to produce a functional truncated protein. The MP11ir isoform is insensitive to Aux/IAA repression. It was also proposed that MP11ir could work independently of auxin. The ectopic expression of *MP11ir* partially complements the *mp/arf5* mutant phenotypes during reproductive development, suggesting that certain MP/ARF5 functions bypass its interaction with Aux/IAA repressors. Coherently, MP11ir activates its target genes in regions of the ovule that are characterized by low auxin concentrations (Cucinotta *et al.*, 2021).

Summing up, despite the structure of the MP/ARF5 protein being known, details on how MP/ARF5 functions *in vivo* are still being uncovered. The detailed role of MR and PB1 domains in forming different protein complexes [MP/ARF5–Aux/IAAs; MP/ARF5–ARFs heterodimerization and oligomerization; MP/ARF5–TFs and MP/ARF5–chromatin remodelers (discussed below)] are continually being discovered. What other post-transcriptional modifications does the MP/ARF5 protein undergo, and how does it affect its stability and activity? These questions remain open. Additionally, the role of the MP/ARF5 isoforms in plant developmental processes is puzzling.

## MP/ARF5-dependent regulation of gene expression

**Fig. 2.**
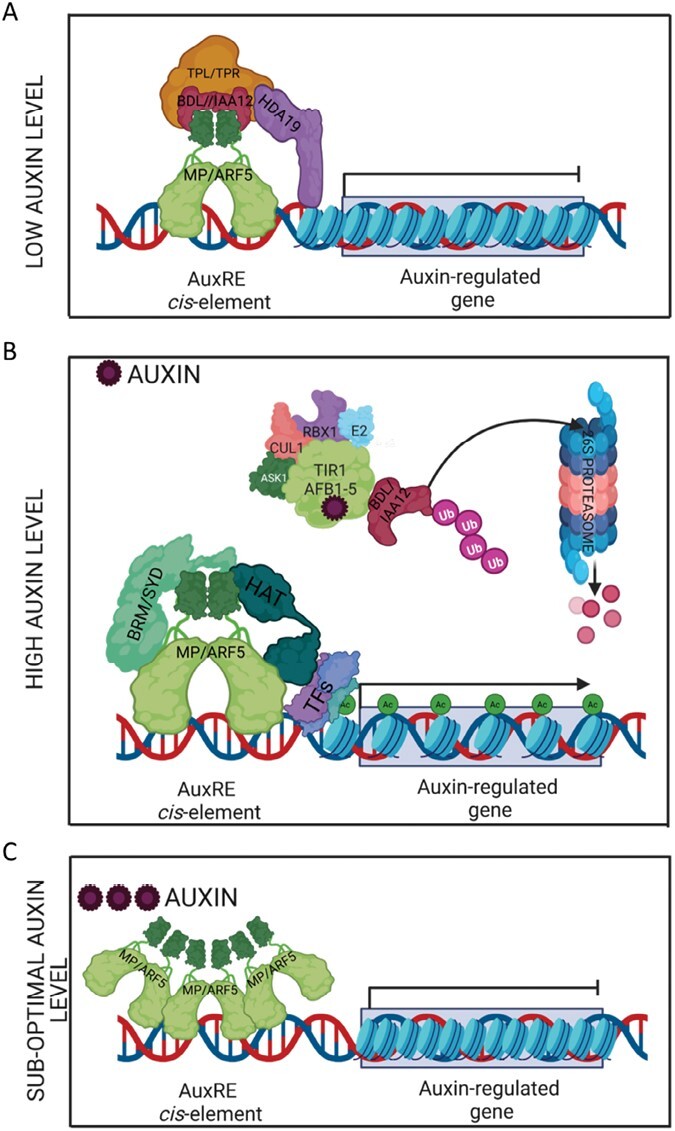
Mechanism of nuclear auxin signaling pathway and auxin-dependent regulation of gene expression by MP/ARF5. BDL/IAA12 repressor binds to MP/ARF5 transcription factor at low cellular auxin levels. BDL/IAA12 repressor recruits co-repressors: TPL/TPRs. Additionally, HDA19 maintains chromatin in an unlicensed repressive state. This interaction prevents MP/ARF5 from driving gene transcription (A). At high cellular auxin concentrations, the hormone is perceived by the TIR1–BDL/IAA12 co-receptor complex, followed by ubiquitination and proteasomal degradation of the BDL/IAA12 protein. The chromatin-remodeling complex containing BRM, SYD, histone acetylases, and other TFs physically associates with MP/ARF5. It facilitates chromatin opening and further activation of gene transcription in response to auxin (B). At a suboptimal auxin level, MP/ARF5 may oligomerize with MP/ARF5 or other ARFs, which can shut down expression of the target genes (C). Ac, acetyl group; ASK1, ARABIDOPSIS SKP1 HOMOLOG; Aux/IAA, AUXIN/INDOLE-3-ACETIC ACID; BDL/IAA12, BODENLOS/INDOLE-3-ACETIC ACID 12; BRM/SYD, BRAHMA/SPLAYED; CUL1, CULLIN 1; E2, UBIQUITIN-LIGASE; HAT, HISTONE ACETYLASE; HDA19, HISTONE DEACETYLASE 19; MP/ARF5, MONOPTEROS/AUXIN RESPONSE FACTOR 5; RBX1, RING-BOX 1; TF, transcription factor; TIR1/AFB, TRANSPORT INHIBITOR RESISTANT1/AUXIN SIGNALING F-BOX; TPL/TPR, TOPLESS/TOPLESS RELATED; Ub, ubiquitin. Created with BioRender.com.

The AuxRE motif recognized by MP/ARF5 is found in the promoter of 11 092 genes and MP/ARF5 protein directly regulates the expression of 4585 target genes in an auxin-dependent way (Xie *et al.*, 2022, Preprint). However, it is essential to note that the molecular mechanisms carried out by MP/ARF5 vary depending on the stages of plant development. It was postulated that in low cellular auxin levels, MP/ARF5 interacts with Aux/IAA repressors (and negative regulators of auxin signaling), mainly IAA12, known as BODENLOS (BDL). MP/ARF5–BDL/IAA12 interaction occurs via their respective PB1 domains. BDL/IAA12 recruits the transcriptional co-repressors TOPLESS (TPL) and TOPLESS-RELATED (TPR), which interact with the histone deacetylase enzyme HDA19 (Long *et al.*, 2006). HDA19 removes the acetyl group from the histone H3 and H4 tails, resulting in condensed chromatin, and the expression of auxin-responsive genes is repressed (Hollender and Liu, 2008) (Fig. 2A).

In the presence of auxin, BDL/IAA12 is recruited by the SCF^TIR1/AFB^ complex, ubiquitinated, and degraded by the 26S proteasome. In the absence of BDL/IAA12, MP/ARF5 recruits chromatin-remodeling complexes containing the BRAHMA (BRM) or SPLAYED (SYD) SWI/SNF ATPases via its MR domain, and this unlocks chromatin through nucleosome eviction and histone acetylation (Wu *et al*., 2015). MP/ARF5 can also interact with the bZIP11 TF, which binds to the G-box-related *cis*-element motif in the vicinity of AuxREs. The bZIP11 TF can recruit the multiprotein SAGA complex (SPT–ADA–GCN5–ACETYLTRANSFERASE), which acetylates nearby histones to open up the chromatin and allow gene transcription by RNA polymerase II (Pol II) (Weiste and Dröge-Laser, 2014; Wu *et al.*, 2015) (Fig. 2B).

Furthermore, it could not be excluded that when cellular auxin levels are at suboptimal concentrations, MP/ARF5 protein might oligomerize with itself or other ARFs, inhibiting the expression of auxin-responsive genes (Fig. 2C). The formation of heterodimers between ARF proteins (i.e. MP/ARF5–ARF1, MP/ARF5–NPH4/ARF7) was experimentally demonstrated (Vernoux *et al.*, 2011). The ARF–ARF oligomerization was observed *in vitro* (Korasick *et al.*, 2014; Nanao *et al.*, 2014) and *in vivo* for ARF19 (Powers *et al.*, 2019). This MP/ARF5–ARF oligomerization scenario is plausible. Indeed, plants with *MP/ARF5* constitutive overexpression (Hardtke *et al.*, 2004) display a pin-like phenotype—a naked inflorescence—similar to the phenotypes of the *mp/arf5* mutant (Przemeck *et al.*, 1996).

## Truncated MPΔ proteins

**Fig. 3.**
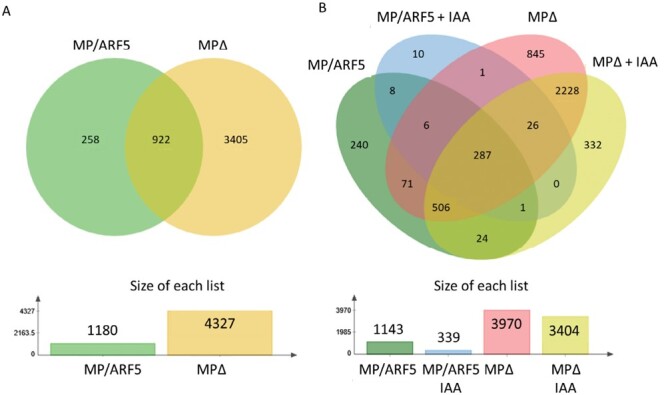
Set of genes directly regulated by canonical MP/ARF5 and truncated MPΔ *in vivo*. Venn diagram showing the overlap between canonical MP/ARF5 and truncated MPΔ gene targets (A). The number of genes under direct MP/ARF5 or MPΔ regulation in response to auxin (B). Venn diagram created with the jvenn tool (Bardou *et al.*, 2014).

To elucidate the complex action of MP/ARF5 and its MP11ir isoform, lines expressing truncated MPΔ proteins (Table 1) may be a powerful tool. These transgenic lines revealed novel MP/ARF5 functions and may help to dissect the functional relevance of MP/ARF5–ARF or MP/ARF5–Aux/IAA interactions. Lines with a truncated MP/ARF5 protein lack the whole PB1 domain (III and IV) or only domain IV, like MPΔ or *mp*^*abn*^, respectively (Garrett *et al.*, 2012; Krogan *et al.*, 2012). The truncated MPΔ protein is presumably not repressible by its Aux/IAA partners, mainly BDL/IAA12, which results in an auxin-independent and enhanced expression of the known MP/ARF5 target genes. The truncated proteins MPΔ and MP^abn^ partially rescue the *mp/arf5* phenotypic defects but also cause leaf defects that were not previously associated with either *mp/arf5* loss- or gain-of-function mutations (Garrett *et al.*, 2012; Krogan *et al.*, 2012). Additionally, it was shown that the MPΔ protein stimulated the ectopic initiation of new organs, specifically in the peripheral zone of the apical meristem (Ma *et al.*, 2019). Interestingly, during *in vitro* culture, MPΔ significantly increases the frequencies of *de novo* shoot formation in Arabidopsis tissue and may be used to overcome organogenic recalcitrance from recalcitrant explants and species (Ckurshumova and Berleth, 2015; Gonzalez *et al.*, 2021). A recent study identified the target genes of MP/ARF5 and truncated MPΔ proteins in 3-day-old seedlings (un)treated with auxin (Xie *et al.*, 2022, Preprint). Only 922 gene promoters out of 4585 were commonly bound by both proteins (Fig. 3A). While MP/ARF5 specifically binds to the promotor of 258 genes, MPΔ interacted with a much higher number of promoters (3405). Interestingly, IAA treatment affected the binding profiles of both proteins differently. In the presence of IAA, 339 target genes were bound by MP/ARF5 (1143 genes in the absence of IAA). For 302 genes out of 339, the interaction was insensitive to the presence of IAA (MP/ARF5 target genes with and without IAA treatment). In contrast, 3404 genes were bound by MPΔ in the presence of IAA (3970 without IAA), among which the interaction was auxin insensitive for 3047 genes (Fig. 3B). This ChIP-seq analysis indicates that the deletion of the PB1 domain modifies the binding profile of MP/ARF5 and renders the protein auxin insensitive. Moreover, differences in the AuxRE motifs preferentially bound by MP/ARF5 and MPΔ suggest that the presence of the PB1 domain may affect homodimer formation and binding to DNA (Xie *et al.*, 2022, Preprint). It raises the possibility that the action of MP/ARF5 and MPΔ may differ at the molecular level and, therefore, differentially impact plant development. An unanswered question is whether the absence of the PB1 domain affects MP/ARF5 interaction with other proteins: ARFs, TFs, and chromatin remodelers. Does the absence of the PB1 domain affect the protein structure and formation of homodimers, heterodimers, and oligomers? In light of research outcomes showing that the PB1 domain is involved in the efficient binding of ARF protein to DNA (Fontana *et al.*, 2022), it would be interesting to investigate how the lack of the PB1 domain in MPΔ affects *cis*-element recognition in the promoter of controlled genes. Does MP/ARF5, like ETT/ARF3, possess an IAA-binding domain and can non-canonically regulate gene expression? Answering those questions would require further studies.

**Table 1.** Genetic material to study MP/ARF5 PB1 domain function.

Line name	Characterization	References
**Chemical and insertional mutants**		
*mp* ^*abn*^ chemical mutagenesis	A nonsense codon at position 837 corresponds to the *mp*^*abn*^ mutation.	Garrett *et al.* (2012)
*mp-14* (SALK_001058) insertional mutagenesis	SALK_001058 has a T-DNA insertion in the 11th exon of MP at the beginning of the sequence coding for the C-terminal domain. Mutant plants have completely penetrant defects only in some developmental processes that depend on MP.	Odat *et al.* (2014)
**Transgenic line**		
*MPΔ* *MPΔ-2* *MPΔ-3*	Driven by the endogenous *MP* promoter (3.3 kb upstream of the start codon). *MPΔ* encodes amino acids 1–813 of MP, followed by a cloning artifact of 20 extra amino acids (DLEELARISPIVQTFGNKVS). *MPΔ-2* and *MPΔ-3* encode amino acids 1–794 and 1–813 of MP, respectively, and lack extra residues.	Krogan *et al.* (2012)
*pMP::MP*Δ *pAS1::MP*Δ	*pMP::MPΔ* contains the endogenous *MP* promoter (3302 kb upstream of the start codon) and the endogenous *MP* transcriptional sequence truncated 730 bp downstream of the stop codon. *MPΔ* encodes amino acids 1–813 of MP without extra residues. In *pAS2::MPΔ*, *MPΔ* is controlled by the *AS2* promoter containing 3303 bp upstream of the *AS2* start codon and 18 bp of the N-terminal *AS2* coding region.	Qi *et al.* (2014)
*pUBQ10::MP794-YPet*	A 794 amino acid truncated version of MP cDNA was amplified with primer set 648f/675r as a translational fusion to C-terminal YPet to generate UBQ10>> MP794-YPet.	Bhatia *et al.* (2016)
*pMP::MP∆-GR* *pMP::MP∆-EAR-GR*	To construct *pMP::MP∆-GR*, a 6695 bp *MP* genomic DNA fragment (containing 3231 bp upstream and 3461 bp downstream of the start codon, respectively) was amplified and fused in-frame to GR. To construct *pMP::MP∆-EAR-GR*, a 120 bp fragment coding for amino acids 183–222 of *AtERF4* was amplified and fused to the C-terminus of *pMP::MP∆.*	Guan *et al.* (2017)
*pPXY::GR-ARF5ΔIII/IV* *p35S::ARF5ΔIII/IV*	To generate *pPXY::GR-ARF5ΔIII/IV* (*pKB25*), the *GR-ARF5ΔIII/IV* fragment, including a stop codon, was amplified from *pKB17* using the MP_for18/MP_rev16 primer pair and inserted in *pTOM50* using *Nco*I/*Cfr*9I restriction sites. To generate *p35S::ARF5ΔIII/IV* (*pKB40*), the *ARF5ΔIII/IV* CDS was amplified from *pKB25* using MP_for17/MP_rev15 and introduced into *pGreen0229-35S* using *Xba*I/*Eco*RI restriction sites.	Brackmann *et al.* (2018)
*pHMG::MP∆*	The *pHMG* promoter corresponds to a 1347 bp fragment upstream of the At1g76110 locus. To generate *MPΔ*, a fragment of the *MP* cDNA coding for a truncated protein before domain III (amino acids 1–794) was amplified.	Ma *et al.* (2019)
*XVE::ARF5Δ DR5rev::GFP*	The first 2382 nucleotides of *MP/ARF5* (encoding the first 794 amino acids and lacking the C-terminal PB1 domain) were cloned into the β-estradiol-inducible, gateway-compatible vector *pMDC7*. This vector was used to transform plants carrying the *DR5rev::GFP* reporter.	Gonzalez *et al.* (2021)
*pUBQ10::MPΔ-TagRFP*	*pMOA34-pUBQ10-loxP-GUS-35S-polyA-loxP-MPΔ-TagRFP* construct contains a 2389 bp *UBQ10* promoter fragment up to the start codon and a 3461 bp genomic fragment for the *MP* coding region.	Guan *et al.* (2022)
**Other approaches**		
*GD-ARF5M* Protoplast transfection assays	The first and last amino acids from MP/ARF5 in *ARF5M*, were 349 and 766.	Tiwari *et al.* (2003)
*MPΔC* Y3H/BiFC	To generate estradiol-inducible *MP*, a truncated version of MP was missing the C-terminal PB1 domain (amino acids 795–902) and was amplified from cDNA, cloned into *pENTR/D-TOPO* (Thermo Fisher). The clone was shuttled into the estradiol-inducible expression vector *pMDC7*.	Wu *et al.* (2015)
*MP*_*ΔIII,IV*_ *GR::MP*_*ΔIII,IV*_ Protoplast transfections Dual-luciferase reporter assay system	Truncated MP protein lacking domains III and IV.	Lau *et al.* (2011) Herud *et al.* (2016)
*pUAS::MPΔ-GUS* *p35S::lox-MPΔ-YFP* *pUBQ10::XVE-amiMP-3AT*	*pSm43GW-pUAS::MPΔ-GUS-OcsT* [OS 30.1] combines *pENTR41R-6xUAS2*, *p221z-cMPdelta*, *p2R3e-GUS-OcsT* into *pSm43GW.* *pBm43GW-p35S::lox-MPΔ-YFP* [OS 74.1] combines *p1R4z-p35S:lox*, *p221z-cMPdelta*, *p2R3a-venYFP-3AT* into *pBm43GW.* *pBm43GW-pUBQ10::XVE-amiMP-3AT* [RM25.1] combines *p1R4a-pUBQ10::XVE*, *pENTR-AtMIR167a-MP*; *p2R3a-3AT* into *pBm43GW*	Smetana *et al.* (2019)

A list of mutants after chemical mutagenesis, insertional mutagenesis, and transgenic lines used to study the truncated MP/ARF5 protein, which acts independently on Aux/IAA repressors.

## Role of the MP/ARF5 transcription factor during the developmental processes

Molecular genetics and cell biological studies have revealed the involvement of MP/ARF5 in several aspects of plant development *in vivo* and *in vitro*, mainly in the model plant Arabidopsis, with the help of numerous tools and transgenic lines (Table 2). This section will illustrate how MP/ARF5 controls genes involved in zygotic embryogenesis, meristem establishment, development of various plant organs, and vascularization patterns through subsequent transcriptional steps.

**Table 2.** Mutants after chemical mutagenesis, insertional mutagenesis, and transgenic lines used to study the canonical MP/ARF5 protein.

Tool	Line name	References
Chemical and insertional mutants	*arf5-1 SALK_023812*	Okushima *et al.* (2005)
	*arf5-2 (mpS319) SALK_021319*	Donner *et al.* (2009)
	*mp-11 SAIL_1265_F06*	Odat *et al.* (2014)
	*mp-12 SALK_149553*	Odat *et al.* (2014)
	*mp-13 WiscDsLoxHs148_11H*	Odat *et al.* (2014)
	*WiscDsLox489-492C10*	Odat *et al.* (2014)
	*SALK_144183*	Odat *et al.* (2014)
	*WiscDsLoxHs148_12G*	Odat *et al.* (2014)
	*mpBS1354*	Hardtke and Berleth (1998)
	*mpG12*	Hardtke and Berleth (1998)
	*mpG92*	Hardtke *et al.* (2004)
	*mpB4149*	Weijers *et al.* (2005)
	*mpBS62*	Vidaurre *et al*. (2007)
Constitutively overexpressed line	*p35S::MP*	Mattsson *et al.* (2003) Hardtke *et al.* (2004)
Inducible lines	*pMP::MP-GR*	Krogan *et al.* (2014)
	*pUBQ10::MP-GR*	Donner *et al.* (2009) Yamaguchi *et al*. (2015)
Silenced lines asRNA or amiRNA	*p35S::MPAS*	Hardtke *et al*. (2004)
	*pMP::amiRARFMP*	Z. Liu *et al*. (2018)
	*pUBQ10::XVE>>amiMP*	Smetana *et al.* (2019)
Transcriptional reporter lines	*pMP::n3×GFP*	Schlereth *et al.* (2010) Rademacher *et al*. (2012)
	*pMP::erYFP*	Smetana *et al.* (2019)
Translational reporter lines	*pMP::MP-GUS*	Vidaurre *et al.* (2007) Krogan *et al.* (2012) Krogan and Berleth (2012) Krogan *et al.* (2014) Carey and Krogan (2017)
	*pMP::MP-GFP*	Cole *et al.* (2009) Schlereth *et al.* (2010) Krogan *et al*. (2012)
	*pMP::MP-YFP*	Bhatia *et al.* (2016)
	*pMP::MP:ECFP*	Donner *et al.* (2009)
Tag lines	*pMP::6×HA*	Weijers *et al.* (2006)
	*pMP::MP-HA*	Krogan *et al.* (2014)
	*pMP::MP-6×HA*	Wu *et al.* (2015)

### Involvement of MP/ARF5 in zygotic embryogenesis

**Fig. 4.**
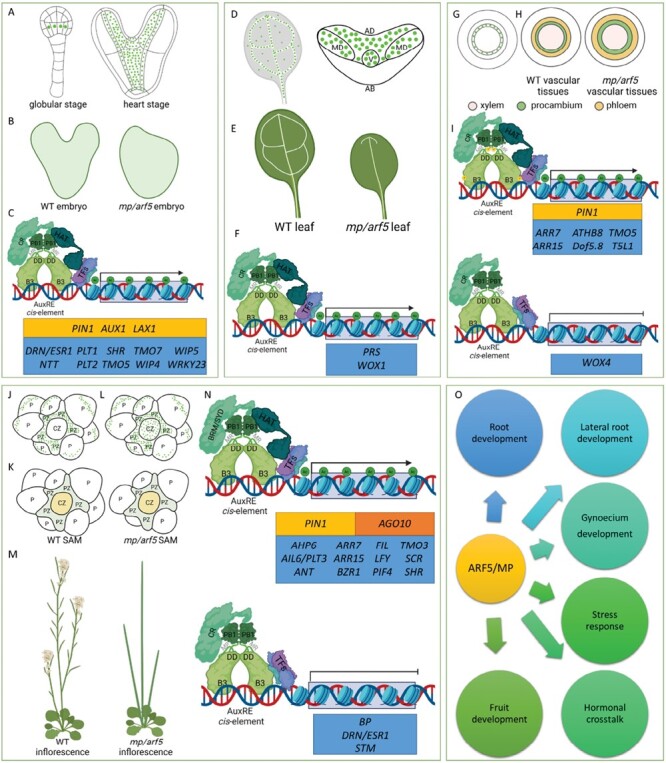
Molecular mechanism of MP/ARF5 action during the developmental process. *MP/ARF5* expression pattern during embryo development in the globular and heart stage (A). The phenotype of the *mp/arf5* mutant in comparison with the wild type (B). Set of MP/ARF5-controlled genes during embryogenesis (C). Localization of MP/ARF5 expression during post-embryogenic leaf development (D). Effect of MP/ARF5 mutation on leaf development (E). Genes under MP/ARF5 control during leaf formation (F). MP/ARF5 activity in the post-embryogenic process of vascularization (G). Effect of MP/ARF5 mutation on the structure of vascular tissues (H). MP/ARF5 controlled genes during canalization (I). Expression pattern of MP/ARF5 during SAM (J) and inflorescence (L) development. Structure of the SAM in the *mp/arf5* mutant in comparison with the wild type (K). Inflorescence of wild-type plants with developed flowers and naked inflorescence of the *mp/arf5* mutant (M). Genes regulated by MP/ARF5 during the formation of meristems. Other processes under MP/ARF5 control (O). Yellow box, genes coding auxin transporters; brown box, gene involved in miR165/166 activity inhibition; blue box, genes coding transcription factors. AB, abaxial domain; Ac, acetyl group; AD, adaxial domain; B3, DNA-binding domain; BRM/SYD, BRAHMA/SPLAYED; CR, chromatin remodeler; CZ, central zone; DD, dimerization domain; HAT, HISTONE ACETYLASES; MD, middle domain; MP/ARF5, MONOPTEROS/AUXIN RESPONSE FACTOR 5; P, phosphoryl group; PB1, Phox/Bem1 domain; P, primordium; PZ, peripheral zone; SAM, shoot apical meristem; TF, transcription factor; V, vascular tissue; WT, wild type. Created with BioRender.com.

MP/ARF5 is essential for development of the female reproductive organ (gynoecium), as shown by the *mp/arf5* mutant displaying smaller gynoecia with a reduction of the stigmatic, stylar, and ovary tissues (Okada *et al.*, 1991; Przemeck *et al.*, 1996; Hardtke and Berleth 1998; Benkova *et al.*, 2003; Bencivenga *et al.*, 2012). The phenotype is more dramatic in *arf3 arf5* double mutants (Pekker *et al.*, 2005), with a near complete loss of ovary tissues. After sexual reproduction and during zygotic embryogenesis, *MP/ARF5* is expressed in the lower tier of the globular embryo (Fig. 4A). *MP/ARF5* transcripts and MP/ARF5 proteins are broadly expressed in the vascular and ground tissue embryonic cells. Subsequently, at the heart stage, *MP/ARF5* is transcribed in the cells of the adaxial side of the cotyledons, the SAM, the quiescent center, the 5–10 fast-dividing cells above the quiescent center, and in subdomains of the vascular tissue (Fig. 4A) (Cole *et al*., 2009; Rademacher *et al.*, 2012). The *mp/arf5* globular embryos are characterized by the aberrant formation of the hypophysis and the vascular tissue due to defects in the division plan: periclinal rather than anticlinal. It results in a disrupted embryonic body plan (embryos lacking hypocotyl and root, and, in the strong mutant alleles, with a single cotyledon) (Fig. 4B) (Mayer *et al.*, 1991; Möller *et al.*, 2017). The MP/ARF5 protein controls the expression of numerous genes involved in embryonic divisions and cell specification (Fig. 4C). The embryonic root initiation process greatly depends on the antagonistic interaction between MP/ARF5 and its repressor BDL/IAA12 protein (Hamann *et al.*, 2002; Schlereth *et al.*, 2010), and both the loss-of-function *mp/arf5* and gain-of-function *bdl/iaa12* mutants are defective in hypophysis specification (Hamann *et al.*, 1999). Interestingly, MP/ARF5 and BDL/IAA12 proteins accumulate in embryonic cells (Weijers *et al.*, 2006; Schlereth *et al.*, 2010), but neither accumulates in the hypophysis, implying that MP/ARF5 drives the hypophysis specification non-cell-autonomously (Hamann *et al.*, 2002; Weijers *et al.*, 2006). The embryonic root initiation also depends on auxin production and transport. Auxin synthesis occurs in the upper tier protoderm of the globular embryo, followed by its transport by the auxin efflux carrier PIN-FORMED 1 (PIN1) and auxin influx carriers AUX1 and LAX2 to the ground tissue, where auxin levels increase (Friml *et al.*, 2003; Robert *et al.*, 2013, 2015; Wabnik *et al.*, 2013). Auxin induces the degradation of the BDL/IAA12 protein, hence promoting the MP/ARF5 activity (Weijers *et al.*, 2006; Schlereth *et al.*, 2010; Herud *et al.*, 2016) to induce the transport of auxin, through the controlled PIN1, AUX1, and LAX2, to the hypophysis precursor (Weijers *et al.*, 2006; Schlereth *et al.*, 2010; Robert *et al.*, 2013, 2015). In the provascular tissue, MP/ARF5 transcriptionally controls the activity of the TFs TARGET OF MONOPTEROS 5 (TMO5) and TMO7, which are critical for embryonic root initiation and formation (Schlereth *et al.*, 2010; Möller *et al.*, 2017). TMO5 and TMO7 belong to the basic helix–loop–helix (bHLH) family and are active in the hypophysis-adjacent embryo cells. TMO5 functions cell autonomously and dimerizes with the bHLH TF LONESOME HIGHWAY (LHW). Together, they regulate periclinal cell divisions, vascular initial cell production, vascular cell proliferation, and xylem fate determination (De Rybel *et al.*, 2013). TMO7 was discovered to be mobile, from the embryo to the uppermost suspensor cell (Schlereth *et al.*, 2010). It contributes to root formation by regulating the asymmetric hypophysis cell division to give rise to the quiescent center and columella cells.

AP2 domain-containing TFs PLETHORA 1 (PLT1) and PLT2 are redundantly required for correct quiescent center specification, leading to the maintenance of the stem cells in the root meristem (Aida *et al.*, 2004). Their expression in the lower tier embryo requires MP/ARF5 and NPH4/ARF7. The *SHORT ROOT* (*SHR*) gene expressed in the provascular cells encodes a mobile TF promoting asymmetric division within the ground tissue, giving rise to root endodermis and cortex (Moreno-Risueno *et al.*, 2015). *SHR* also requires the transcriptional activity of MP/ARF5 (Möller *et al.*, 2017). Genes encoding the zinc finger TFs, NO TRANSMITTING TRACT (NTT), and its paralogs WIP DOMAIN PROTEIN 4 (WIP4) and WIP5, are the targets of MP/ARF5 and required for root meristem initiation (Crawford *et al.*, 2015). Likewise, WRKY23 acts downstream of ARF5–BDL/IAA12 during embryogenesis and formation of the hypophysis. Moreover, expression patterns of *MP/ARF5*, *BDL/IAA12*, and *WRKY23* overlap in the inner cells of young embryos, excluding the protoderm. The *pWRKY23::GUS* activity in the inner cells of a 16-cell stage embryo was absent in the gain-of-function (auxin-resistant) *bdl/iaa12* embryos. Additionally, the morphological defects observed in the *wrky23* mutant are very similar to those seen in *mp/arf5* and *bdl/iaa12* mutants (Grunewald *et al.*, 2013).

Many genes linked to embryogenic root formation were documented to be a direct downstream target of the MP/ARF5. Also, in post-embryonic development, MP/ARF5 is involved in root formation. MP/ARF5 directly regulates the expression of *MIR390* in the primary root meristem (Dastidar *et al.*, 2019); miR390 triggers the biogenesis of 21 nucleotide secondary siRNAs called ta-siRNAs targeting *ARF2*, *ETT/ARF3*, and *ARF4* transcripts (Marin *et al.*, 2010). MP/ARF5 is also expressed during lateral root founder cell specification, nuclear migration, and lateral root initiation processes. *ETHYLENE RESPONSE FACTOR 114* (*ERF114*) and *ERF115* activate auxin signaling via induction of *MP/ARF5* and promote lateral root development (Canher *et al.*, 2020, 2022). In dividing pericycle cells, the BDL/IAA12–MP/ARF5-mediated auxin response guarantees an organized lateral root patterning downstream of SOLITARY-ROOT (SLR)/IAA14 (de Smet *et al.*, 2010).

Less is known about the role of MP/ARF5 in SAM and cotyledon formation during embryogenesis. MP/ARF5 controls the expression of the *DORNRÖSCHEN*/*ENHANCER OF SHOOT REGENERATION 1* (*DRN*/*ESR1*) gene, encoding an AP2 transcription factor involved in embryonic patterning, auxin perception, and transport (Chandler *et al.*, 2008; Cole *et al.*, 2009). Based on ChIP and phenotypic analysis, it was demonstrated that MP/ARF5 positively regulates *DRN/ESR1* via binding to two AuxRE motifs in its promoter. This genetic interaction between MP/ARF5 and *DRN/ESR1* is also essential for DRN/ESR1 to contribute to the development of embryonic cotyledons (Cole *et al.*, 2009). These results align with studies that showed a lack of *pDRN::GFP* activity in the cotyledon tips of the *mp/arf5* insertional mutant (Okushima *et al.*, 2005). The role of ARF5 in fruit development has been confirmed in tomatoes, where *SlARF5* plays a role in fruit initiation and is associated with auxin/gibberellic acid crosstalk (S. Liu *et al*., 2018), and the members of the *LcARF5A/B* clade may promote fruit abscission in litchi (Zhang *et al.*, 2019). In apples, MdARF5 directly binds to the promoters of ethylene biosynthetic genes *MdACS3a*, *MdACS1*, and *MdACO1* to induce their expression, initiating fruit ripening (Yue *et al.*, 2020).

Polar auxin transport, properly organized cell divisions, cell specification, and intercellular communication are required to form the zygotic embryo—MP/ARF5 appears to coordinate all these aspects.

### Leaf formation and establishment of its abaxial–adaxial polarity

During embryogenic development, MP/ARF5 governs the formation of embryo apical–basal polarity. MP/ARF5 is also responsible for establishing organ polarity during post-embryonic development. We can distinguish three domains in a leaf: adaxial, abaxial, and, on the borderline, middle domains. The adaxial domain has lower auxin levels than the abaxial, leading to a specific high auxin response in the middle domain (Qi *et al.*, 2014; Guan *et al.*, 2017; Burian *et al.*, 2022). In early leaf developmental stages, *MP/ARF5* is expressed in leaf primordia and most leaf regions (Hardtke *et al.*, 2004; Wenzel *et al.*, 2007; Krogan and Berleth, 2012; Schuetz *et al.*, 2019; Burian *et al.*, 2022). The initial broad expression domain of MP/ARF5 is gradually restricted to the leaf veins, and adaxial and middle domains (Pekker *et al.*, 2005; Guan *et al.*, 2017) (Fig. 4D). The Arabidopsis *mp/arf5* and tomato *slarf5* mutant leaf blades have a reduced surface area (Fig. 4E) (Israeli *et al.*, 2019; Schuetz *et al.*, 2019), and both *arf3 arf5* and *arf5 arf7* double mutants could not form leaves and displayed enlarged meristems, indicating that MP/ARF5 functions synergistically with ETT/ARF3 and NPH4/ARF7 (Schuetz *et al.*, 2019). The adaxial–abaxial polarity is altered in the leaves of the gain-of-function mutant MPΔ. The mutant displays an irregular and narrow leaf shape due to restricted laminar expansion, disrupted adaxial–abaxial polarity, and vasculature hypertrophy, compared with the hypotrophy observed in *mp/arf5* loss-of-function mutants. It implies that MP/ARF5 plays a prominent role in establishing organ polarity (Krogan and Berleth, 2012). MP/ARF5 directly activates the expression of *WUSCHEL-RELATED HOMEOBOX 1* (*WOX1*) and *PRESSED FLOWER* (*PRS*) in the middle domain, leading to an outgrowth of the leaf (Guan *et al.*, 2017) (Fig. 4F).

### Vascular tissue formation

*MP/ARF5* is strongly expressed in pre-vascular, pre-procambial, procambial, and cambial cells (Fig. 4G), but less abundantly in the already differentiated tissues, namely the phloem and xylem (Schuetz *et al.*, 2019; Agustí and Blázquez, 2020). MP/ARF5 activity is crucial for embryonic and post-embryonic vascular tissue development. The *mp/arf5* mutant embryos have an incomplete vascular system and are defective in the formation of the root and the embryonic body axis (Hamann *et al.*, 1999; Hobbie *et al.*, 2000). The mutant embryos also have fewer cell files in the vascular tissue (Fig. 4H) (De Rybel *et al.*, 2013). MP/ARF5 is implicated in leaf vein patterning and development (Fig. 4I). *MP/ARF5* and *PIN1* expression patterns overlapped in pre-procambial veins. In addition, their respective mutants have leaf vascular defects (Wenzel *et al.*, 2007; Bhatia *et al.*, 2016; Krogan *et al.*, 2016). MP/ARF5 also positively and directly regulates the expression of the HD-ZIPIII gene *ARABIDOPSIS THALIANA HOMEOBOX GENE 8* (*ATHB8*) through binding to the AuxREs in its promoter. ATHB8 is involved in leaf pre-procambial cell fate acquisition (Donner *et al.*, 2009). Similarly, in the woody species *Populus tomentosa*, PtoIAA9 interacts with PtoARF5 to form a PtoIAA9–PtoARF5 module that directly controls the expression of the homeobox *PtoHB7* and *PtoHB8* genes, encoding HD-ZIPIII TFs responsible for mediating the secondary xylem development (Xu *et al.*, 2019).

During embryogenesis, TMO5 and its closest homolog, TMO5-LIKE1 (T5L1), play a pivotal role in vascular development in roots. Both genes are expressed in an MP/ARF5-dependent manner in the xylem precursor cells of the root and the vasculature of the globular embryo (Schlereth *et al.*, 2010; De Rybel *et al.*, 2013). The mature embryos and seedlings of the *tmo5 t5l1* double mutant display severe defects in the vascular tissue, proving that MP/ARF5 mediates vascular tissue establishment during embryogenesis through its two direct target genes, *TMO5* and *T5L1* (De Rybel *et al.*, 2013). The heterodimer TMO5–LHW induces the expression of *LONELY GUY 3* (*LOG3*) and *LOG4*, encoding cytokinin biosynthetic enzymes. Cytokinin is required for the periclinal cell divisions within procambial cells. MP/ARF5 can also induce the expression of the *Dof5.8* gene in the pre-procambial stage (Konishi *et al.*, 2015). MP/ARF5 is post-transcriptionally modified during the vascularization process by BIN2-LIKE 1 (BIL1), a glycogen synthase kinase. BIL1 phosphorylates MP/ARF5 and enhances its activity in regulating the expression of *ARABIDOPSIS RESPONSE REGULATOR* (*ARR*) genes, *ARR7* and *ARR715.* ARR7 and ARR15 are negative regulators of the cytokinin response, leading to inhibition of cambial activity (Han *et al.*, 2018). Additionally, MP/ARF5 predominantly promotes xylem production by directly activating xylem-related genes and repressing *WOX4* (Brackmann *et al.* 2018). However, the conditional knockdown of *MP/ARF5* also leads to the down-regulation of *WOX4* (Smetana *et al.*, 2019), questioning the molecular mechanisms by which MP/ARF5 regulates *WOX4.*

### Shoot apical meristem and inflorescence development

The MP/ARF5 protein controls embryonic and post-embryonic SAM development. MP/ARF5 accumulates in both differentiated and undifferentiated SAM cells (Hardtke and Berleth, 1998; Schlereth *et al.*, 2010; Zhao *et al.*, 2010), specifically in the peripheral zone and in the developing primordia, but not in the central zone (Fig. 4J) (Burian *et al.*, 2022). The *mp/arf5* mutant initiates rosette leaves at a slightly reduced rate (Fig. 4K) (Bennett *et al.*, 1996; Przemeck *et al.*, 1996; Gälweiler *et al.*, 1998). The development of axillary branches necessitates stem cell maintenance, axillary meristem initiation, differentiation, and axillary bud outgrowth. Axillary meristems are formed from a small number of stem cells with meristematic competence located in cauline leaf axils. During embryonic and post-embryonic development, ARGONAUTE 10 (AGO10), spatiotemporally controlled by auxin, brassinosteroids, and light, promotes embryo and axillary meristem development through *miR165/166* sequestration, resulting in increased levels of *miR165/166* target transcripts: *PHABULOSA* (*PHB*), *PHAVOLUTA* (*PHV*), and *REVOLUTA* (*REV*). In axils of young leaves, MP/ARF5 activates *BRASSINAZOLE-RESISTANT 1* (*BZR1*) and *PHYTOCHROME-INTERACTING FACTOR 4* (*PIF4*), which directly repress *AGO10* transcription and prevent axillary meristem initiation. In axils of older leaves, MP/ARF5 up-regulates *AGO10* expression to promote axillary meristem initiation (Turchi *et al.*, 2013; Zhang *et al.*, 2020). Interestingly, *MP/ARF5* may be a direct target of PHB, indicating a complex interaction mechanism resembling a feedback loop (Qi *et al.*, 2014; Müller *et al.*, 2016; Guan *et al.*, 2017).

During inflorescence formation, MP/ARF5 accumulates in the central zone, peripheral zone, and the developing flower primordia (Fig. 4L) (Bhatia *et al.*, 2016). MP/ARF5 is crucial for inflorescence development. The *mp/arf5* mutant and plants with a constitutive *MP/ARF5* overexpression give rise to pin-shaped inflorescences (Fig. 4M) (Hardtke *et al.*, 2004; Carey and Krogan, 2017). The MPΔ lines develop flowers, but floral organs, such as petals, are narrow with marked increased vascularization (Krogan and Berleth, 2012). Besides MP/ARF5, loss-of-function mutations in the genes involved in auxin biosynthesis, the auxin efflux carrier *PIN1*, and the AGC kinase *NAKED PINS IN YUC MUTANTS5* (*NPY5*) abolish floral meristem formation, resulting in the naked inflorescence stem phenotype (Reinhardt *et al.*, 2000; Vernoux *et al.*, 2000; Cheng *et al.*, 2006, 2008). These observations indicate that auxin biosynthesis, transport, and signaling are indispensable in floral meristem initiation and inflorescence organization. On the one hand, MP/ARF5 contributes to flower initiation by directly up-regulating the primary floral development regulator genes (Fig. 4N) such as *LEAFY* (*LFY*), *AINTEGUMENTA* (*ANT*), *AINTEGUMENTA-LIKE 6*/*PLETHORA 3* (*AIL6/PLT3*), *FILAMENTOUS FLOWER* (*FIL*), *TMO3/CYTOKININ RESPONSE FACTOR 2* (*CRF2*), *SCARECROW* (*SCR*), and *SHR* (Cole *et al.*, 2009; Krizek, 2009; Yamaguchi *et al.*, 2013; Wu *et al.*, 2015; Bahafid *et al.*, 2022, Preprint). On the other hand, MP/ARF5 monitors flower primordium initiation by contributing to reprogramming cell identities from stem cell descendent to primordium founder cell fate in the inflorescence. Auxin-activated MP/ARF5 recruits proteins from the SWI/SNF chromatin-remodeling complex, which opens the chromatin to the repressing TFs binding the promoter of the essential regulators for flower initiation (Wu *et al.*, 2015). SYD and BRM, two related Arabidopsis SWI/SNF ATPases (Bezhani *et al.*, 2007; Shu *et al.*, 2022), are expressed in flower primordia (Wu *et al.*, 2012). In addition, *brm-3 syd-5* double mutants exhibit pin-like inflorescence phenotypes, indicating their crucial role in initiating flower primordium. Interestingly, ChIP experiments identified SYD and BRM binding to regulatory sites of MP/ARF5 targets in the proximity of the AuxREs bound by MP/ARF5. It suggests that MP/ARF5 recruits this SWI/SNF chromatin-remodeling complex to its target loci to loosen the compacted chromatin and triggers the transcriptional activation of these loci (Wu *et al.*, 2015). Additionally, the down-regulation of the TF genes *SHOOTMERISTEMLESS* (*STM*) and *BREVIPEDICELLUS* (*BP*), key pluripotency genes stimulating meristematic fate, is crucial for reproductive primordium initiation. MP/ARF5 contributes indirectly to *STM* repression by up-regulating FIL. In parallel, MP/ARF5 acts with ETT/ARF3 and ARF4 repressors expressed in incipient reproductive primordia and promoter of flower initiation through histone acetylation (Chung *et al.*, 2019). MP/ARF5 also activates the expression of *ARABIDOPSIS HISTIDINE PHOSPHOTRANSFER PROTEIN 6* (*AHP6*) (Besnard *et al.*, 2014), which encodes a negative regulator of the cytokinin signaling pathway (Mähönen *et al.*, 2006). AHP6 has been shown to non-cell-autonomously suppress meristematic cell activity in the tissue surrounding floral primordia and thus to assist in establishing phyllotaxis, the regular pattern of primordia emergence (Besnard *et al.*, 2014). Notably, it down-regulates, together or in parallel with MP/ARF5 (Zhao *et al.*, 2010), the expression of type-A *ARR* (Chahtane *et al.*, 2013), negatively regulating cytokinin responses. ARR7 and ARR15 are potent regulators of SAM function and negative regulators of cytokinin signaling, and their promoters are targets of MP/ARF5-mediated auxin signaling (Zhao *et al.*, 2010). It was demonstrated that the up-regulation of *SHR* expression at sites of organ initiation depends on auxin, acting through MP/ARF5. In the central zone, the SHR-target SCR-LIKE 23 (SCL23) physically interacts with WUSCHEL (WUS), a key regulator of stem cell maintenance. *SCL23* and *WUS* expression is subject to negative feedback regulation from stem cells through the CLAVATA (CLV) signaling pathway (Bahafid *et al.*, 2022, Preprint). MP/ARF5 mediates auxin signaling responses in the peripheral zone and, in the central zone, binds directly to the promoter of *DRN* and represses its activity (Luo *et al.*, 2018). *DRN* is expressed in the center of the meristem, where it up-regulates *CLV3* expression, suggesting a mechanism where MP/ARF5-mediated auxin signaling controls stem cell activity by regulating *DRN* expression. This provides a scenario where WUS establishes a minimal auxin signaling activity in the central zone. This activity requires MP/ARF5 to fine-tune CLV3 activity, helping to regulate stem cell homeostasis. For organ primordia specification in the peripheral zone, temporal information carried by auxin is essential, as is the repression of *STM* expression by ETT/ARF3, ARF4, and MP/ARF5 auxin signaling effectors (Luo *et al.*, 2018; Chung *et al.*, 2019; Ma *et al.*, 2019).

The general absence of H3K27me3, a hallmark of POLYCOMB REPRESSIVE COMPLEX 2 (PRC2) activity, at the *MP/ARF5* locus indicates that their regulation does not rely on this epigenetic mechanism. Interestingly, the H3K4me3 marks, and accessible regions identified by FANS-ATAC on the *MP/ARF5* promoter, were present, which suggests a constitutively active *MP/ARF5* locus. These properties suggest that the chromatin configuration of the *MP/ARF5* locus allows it to be actively transcribed in different tissues and at different developmental stages. They also imply that the spatial expression pattern specific to the *MP/ARF5* gene does not result primarily from alternative chromatin states with contrasting accessibility. The regulatory system of *MP/ARF5* expression might be based on the transcriptional activators, repressors, and post-translational modifications that modulate the activity of MP/ARF5. A few proteins can potentially regulate *MP/ARF5* expression, namely KNOTTED-LIKE FROM ARABIDOPSIS THALIANA 1 (KNAT1), SCHLAFMUTZE (SMZ), Dof1.8, and LOB DOMAIN-CONTAINING PROTEIN 3 (LBD3). In particular, Dof1.8 may repress *MP/ARF5* expression, as *MP/ARF5* and *DOF1.8* genes show complementary expression patterns (Truskina *et al.*, 2021). It cannot be excluded that *MP/ARF5* may also be regulated during the translation process via the GTPase ROP2–TARGET OF RAPAMYCIN (TOR) pathway. The levels of translation reinitiation of *MP/ARF5* can be increased several fold without affecting mRNA stability (Schepetilnikov and Ryabov, 2017).

### Role of MP/ARF5 in developmental processes *in vitro*

MP/ARF5 is crucial for indirect *de novo* shoot organogenesis, and its expression determines the acquisition of shoot progenitor cell identity (Liu *et al.*, 2022, Preprint). Callus formation is inhibited in the *mp/arf5* mutant, and explants are impaired in producing shoots (Zhang *et al.*, 2021). In contrast, transgenic lines with a dominantly acting variant of MPΔ showed increased regeneration, even from recalcitrant tissues (Ckurshumova *et al.*, 2014; Gonzalez *et al.*, 2021). Interestingly, the MPΔ variant is associated with a higher *WUS* expression in spots where the shoot formation occurred. MP/ARF5 can positively regulate downstream cytokinin response factor *TMO3*/*CRF2* (Ckurshumova *et al.*, 2014). The *crf2* mutation abolishes the formation of SAMs on calli of MPΔ cotyledon explants and diminishes the *WUS* and *STM* expression. In contrast, *TMO3/CRF2* overexpression confers higher shoot-forming properties in a wild-type background. Thus, the shoot-promoting influence of MPΔ is likely to be partially conferred by *TMO3/CRF2*. ARF4 is also involved in shoot regeneration and depends on the presence of MP/ARF5 (Zhang *et al.*, 2021). Additionally, MP/ARF5 is involved in the auxin-dependent induction of somatic embryogenesis. The *MP/ARF5* gene is the most highly up-regulated member of the *ARF* family during somatic embryogenesis induction, and the *mp/arf5* mutant rarely regenerates somatic embryos (Wójcikowska and Gaj, 2017).

### Stress response

ARF5 is involved in abiotic and biotic stress responses. In *Ipomoea batatas*, *IbARF5* regulates salt and drought tolerance, and the overexpression of *IbARF5* up-regulates ABA biosynthetic genes (Kang *et al.*, 2018). In lettuce, the *ARF5* gene was studied to understand the molecular mechanism underlying the phenomenon of early bolting caused by high temperatures and reducing the quality and taste of the lettuce leaf. The relative *ARF5* expression was significantly down-regulated in lettuce varieties susceptible to bolting under high temperatures, suggesting that ARF5 may be involved in vegetable bolting (Pan *et al.*, 2019). The *Osarf5* mutant showed an increased tolerance or enhanced resistance to rice dwarf virus infection, lower disease incidence, and accumulation of rice dwarf virus proteins (Qin *et al.*, 2020). It was demonstrated that auxin inhibits stomatal development through nuclear TIR1/AFB-mediated auxin signaling and that MP/ARF5 binds to the *STOMAGEN* promoter, inhibiting its expression in mesophyll and stopping stomatal development (Zhang *et al.*, 2014).

## Concluding remarks and future perspectives

Among the members of the ARF family, MP/ARF5 is at the center of scientific interest. MP/ARF5 is the key TF regulating almost every aspect of plant development. Research on it contributes knowledge about the regulation of gene expression, particularly in response to auxin. Analytical tools allow the study of the interactions between MP/ARF5 and DNA, as well as MP/ARF5 and other proteins, revealing an extremely complex interaction network. Many genes that may be under the direct control of MP/ARF5 have been recognized. The challenge of the next few years will be to learn how the level of MP/ARF5 as an oligomer, dimer, or monomer affects its function. It will also be essential to discover in detail the activator and repressor regulatory elements acting upstream of MP/ARF5. It is interesting to study epigenetic factors affecting the activity of the *MP/ARF5* locus, especially in the context of recent data indicating that this locus is constantly open (Truskina *et al.*, 2021). Are modifications of DNA and histones or the composition of nucleosomes containing various histone variants responsible for this open chromatin state? This question remains unanswered. When single-cell DNA and histone modification profiling are widely adopted in plants, it will help to uncover how MP/ARF5 is epigenetically regulated. Another open question remains: is MP/ARF5 essential and acting as a key player in plant development? Is *MP/ARF5* unique among other *ARF* genes? Or is there a bias in this statement because ARF5 has been and is the focus of so many studies? Many years of detailed research on the process of zygotic embryogenesis have led to the identification of 510 *EMBRYO-DEFECTIVE* (*EMB*) genes in Arabidopsis involved in the zygotic embryogenic process, whose mutation induces defects during embryogenesis (Meinke, 2020). It seems likely that most genes encoding TFs will play a key role at some stages of plant development *in vivo*. However, numerous studies are still needed to identify the function of all TF genes, especially those acting redundantly. Until then, we can conclude that MP/ARF5 reigns supreme over the other members of the ARFs and most of the genes encoding TFs.

## Abbreviations

ARF: AUXIN RESPONSE FACTOR
ARR: ARABIDOPSIS RESPONSE REGULATOR
Aux/IAA: AUXIN/INDOLE-3-ACETIC ACID
AuxRE: auxin-responsive element
BDL: BODENLOS
BRM: BRAHMA
CRF2: CYTOKININ RESPONSE FACTOR 2
DBD: DNA-binding domain
DD: dimerization domain
DRF: DORNRÖSCHEN
ERF: ETHYLENE RESPONSE FACTOR
ESR1: ENHANCER OF SHOOT REGENERATION 1
ETT: ETTIN
HDA19: HISTONE DEACETYLASE 19
IAA: indole-3-acetic acid
IAM: indolyl-3-acetamide
IAN: indolyl-3-acetonitrile
MP: MONOPTEROS
MR: middle region
NPH4: NON-PHOTOTROPHIC HYPOCOTYL 4
PB1: Phox/Bem1
PIN: PIN-FORMED
SAM: shoot apical meristem
TF: transcription factor
TMO: TARGET OF MONOPTEROS
WUS: WUSCHEL
